# Ginkgolide With Intravenous Alteplase Thrombolysis in Acute Ischemic Stroke Improving Neurological Function: A Multicenter, Cluster-Randomized Trial (GIANT)

**DOI:** 10.3389/fphar.2021.792136

**Published:** 2021-12-03

**Authors:** Xuting Zhang, Wansi Zhong, Xiaodong Ma, Xiaoling Zhang, Hongfang Chen, Zhimin Wang, Min Lou

**Affiliations:** ^1^ Department of Neurology, The Second Affiliated Hospital of Zhejiang University, School of Medicine, Hangzhou, China; ^2^ Department of Neurology, Haiyan People’s Hospital, Jiaxing, China; ^3^ Department of Neurology, The Second Affiliated Hospital of Jiaxing University, Jiaxing, China; ^4^ Department of Neurology, Affiliated Jinhua Hospital, Zhejiang University School of Medicine, Jinhua, China; ^5^ Department of Neurology, The First People’s Hospital of Taizhou, Taizhou, China

**Keywords:** Ginkgolide®, stroke, intravenous alteplase, prognosis, improve

## Abstract

**Background and Purpose:** We aimed to investigate the effect of Ginkgolide® treatment on neurological function in patients receiving intravenous (IV) recombinant tissue plasminogen activator (rt-PA).

**Methods:** This cluster randomized controlled trial included acute ischemic stroke patients in 24 centers randomized to intervention of intravenous Ginkgolide® or control group within the first 24 h after IV rt-PA therapy (IVT). Clinical outcome at 90 days was assessed with modified Rankin Scale (mRS) score and dichotomized into good outcome (0–2) and poor outcome (3–6). Hemorrhagic transformation represented the conversion of a bland infarction into an area of hemorrhage by computed tomography. Symptomatic intracerebral hemorrhage (sICH) was defined as cerebral hemorrhagic transformation in combination with clinical deterioration of National Institutes of Health Stroke Scale (NIHSS) score ≥4 points at 7-day or if the hemorrhage was likely to be the cause of the clinical deterioration. We performed logistic regression analysis and propensity score matching analysis to investigate the impact of Ginkgolide® treatment with IV rt-PA on good outcome, hemorrhagic transformation and sICH, respectively.

**Results:** A total of 1113 patients were finally included and 513 (46.1%) were in the intervention group. Patients in the Ginkgolide® group were more likely to have good outcomes (78.6 vs. 66.7%, *p* < 0.01) and lower rate of sICH (0 vs. 2.72%, *p* < 0.01), compared with patients in the control group. The intra-cluster correlation coefficient (ICC) for good outcome at 90 days was 0.033. Binary logistic regression analysis revealed that treatment with Ginkgolide® was independently associated with 90-day mRS in patients with IV rt-PA therapy (OR 1.498; 95% CI 1.006–2.029, *p* = 0.009). After propensity score matching, conditional logistic regression showed intervention with Ginkgolide® was significantly associated with 90-day good outcome (OR 1.513; 95% CI 1.073–2.132, *p* = 0.018). No significant difference in hemorrhage transformation was seen between the 2 matched cohorts (OR 0.885; 95% CI 0.450–1.741, *p* = 0.724).

**Conclusion:** Using Ginkgolide® within 24-hour after IV rt-PA is effective and safe and might be recommended in combination with rtPA therapy in acute ischemic stroke.

**Clinical Trial Registration:** http://www.clinicaltrials.gov, identifier NCT03772847.

## Introduction

Intravenous recombinant tissue plasminogen activator (rt-PA) administered within 4.5 h of onset (or later if a favorable perfusion imaging profile is present) could improve neurological outcome in patients with acute ischemic stroke (AIS). Treatments for AIS continue to evolve after the superior value of endovascular thrombectomy was confirmed over systemic thrombolysis. Unfortunately, up to 50% of such patients with successful recanalization still have an unfavorable outcome (Hussein et al., 2018), and numerous neuroprotective drugs have failed to show benefit in the treatment of AIS (Zhao et al., 2020). New methods to enhance the general efficacy of intravenous thrombolysis (IVT) are imperative in patients with AIS (Knecht et al., 2018).

The core problem in acute stroke is the loss of neuronal cells which makes recovery difficult or even not possible in the late states. Several key players in neuronal cell death within the penumbra have been identified, including excitotoxicity, oxidative stress, and inflammation. Oxidative stress directly leads to DNA damage that occurs within minutes after cerebral ischemic strokes (Li et al., 2018). Reperfusion therapy accompanying re-entry of oxygen and glucose into the ischemic brain fuels an excess production of reactive oxygen species (ROS) that overwhelms endogenous antioxidant reserves and leads to reperfusion injury. Researchers observed that cerebral ROS generation peaked 1 day after transient middle cerebral artery occlusion (MCAO) in mice, coinciding with an increase in Nrf2, a transcription factor that regulates antioxidant enzymes (Takagi et al., 2014; Yumiko et al., 2017).

Ginkgo biloba leaves extracts can protect against neuronal death caused by ischemia in animal stroke models (Feng et al., 2019). These pharmacological effects are attributed to two major groups of chemical constituents, namely, flavonoids and terpene lactones. Terpene lactones includes ginkgolides A, B, and C, and bilobalide, which are the main components of Ginkgolides®. In MCAO rats, ginkgolides B treatment could significantly increase the expressions of anti-oxidative stress-related proteins, including superoxide dismutase (SOD). Diterpene ginkgolides (ginkgolide A, ginkgolide B and ginkgolide C) were reported to activate Akt signaling and lead to the nuclear location of Nrf2, which has protective effects against oxidative stress (Liu et al., 2019). Furthermore, in Sprague daw rats with MCAO, pretreatment with bilobalide improved neurological function and increased SOD activity while decreasing infarct volume and brain edema (Jiang et al., 2014).

The immune-mediated inflammatory response that follows AIS is a therapeutic target under current investigation. Previous studies have shown that ginkgolides can reduce inflammation, ameliorate the metabolic disturbances caused by rt-PA. A derivate of ginkgolide B named XQ-1H, suppressed neutrophils infiltration and inflammatory mediators, including matrix metalloproterinase-9 in the ischemic region of the brain (Fang et al., 2015). Down-regulated matrix metalloproterinase-9 expression could reduce extracellular matrix degradation and protect blood brain barrier permeability (BBB) via tight junction in brain endothelial cells (Wei et al., 2013). Finally, pre-administration of XQ-1H reduced cerebral infarct size and diminished brain edema after stroke in rats.

Ginkgolide was found to be specific and selective antagonist of platelet activating factor (PAF) (Koch, 2005), which was involved in thrombosis for strong platelet aggregation. Thus, Ginkgolide may enhance the general efficacy of IVT through its antioxidation, anti-inflammatory and antithrombotic mechanisms. But so far, there is no large-scale clinical trial to confirm the general efficacy of Ginkgolide® in AIS with IVT therapy. Moreover, whether Ginkgolide can increase the risk of hemorrhagic transformation after intravenous thrombolysis in AIS patients is unclear. Thus, we aimed to determine the clinical efficacy and safety of Ginkgolide® combined with IV rt-PA in AIS.

## Methods

### Study Design and Participants

GIANT was an open label, prospective, multicenter cluster-randomized clinical trial involving 24 hospitals in China (NCT03772847). We enrolled patients who 1) were 18 years or older; 2) were AIS patients who met the criteria of IVT (William et al., 2019); 3) or his/her family member signed an informed consent. We excluded patients who 1) were diagnosed as cerebral arteritis; 2) with baseline alanine aminotransferase or aspartate aminotransferase ≥3 times the upper limit of normal, or baseline serum creatinine ≥1.5 times the upper limit of normal; 3) were allergic to ginkgo drugs, alcohol or glycerol; 4) participated in other clinical trials. The criteria for intravenous thrombolysis was as follows: 1) Inclusion criteria:Clinical diagnosis of ischemic stroke causing measurable neurologic deficit; Onset of symptoms <4.5 h before beginning treatment; Age ≥18 years; 2) Exclusion criteria: Ischemic stroke or severe head trauma in the previous 3 months; Previous intracranial hemorrhage; Intra-axial intracranial neoplasm; Gastrointestinal malignancy; Gastrointestinal/Urinary hemorrhage in the previous 21 days; Major surgery in the preceding 14 days; Had an arterial puncture of a noncompressible blood vessel in the previous 7 days; Intracranial or intraspinal surgery within the prior 3 months; Symptoms suggestive of subarachnoid hemorrhage; Persistent blood pressure elevation (systolic ≥185 mmHg or diastolic ≥110 mmHg); Glucose levels <50 or >400 mg/dl; Active internal bleeding; Presentation consistent with infective endocarditis; Stroke known or suspected to be associated with aortic arch dissection; Acute bleeding diathesis; Platelet count <100,000/mm^3^; Current anticoagulant use with an INR >1.7 or PT > 15 s or aPTT >40 s; Therapeutic doses of low molecular weight heparin received within 24 h; Current use of a direct thrombin inhibitor or direct factor Xa inhibitor with evidence of anticoagulant effect by laboratory tests such as aPTT, INR, ECT, TT, or appropriate factor Xa activity assays; Evidence of hemorrhage; Extensive regions of obvious hypodensity consistent with irreversible injury.

The human ethics committee of the Second Affiliated Hospital of Zhejiang University (SAHZU), School of Medicine, approved the trial protocol. The clinical trial was conducted according to the principle expressed in the Declaration of Helsinki. Written consent was obtained from patients or their relatives.

### Randomization

The randomization was conducted by using a computer generating randomization sequence where twenty-four hospitals were assigned to the Ginkgolide intervention or control group randomly. Data on all thrombolytic patients in both groups were consecutively recorded in a secure, purpose-built web-based data entry system.

### Interventions

Patients in hospitals which were allocated to the treatment arm received rt-PA (0.9 mg/kg) and an intravenous infusion of Ginkgolide® (10 ml dissolved in a vehicle containing 250 ml normal saline, once a day, continuous intravenous injection for at least 7 days). The recommended course of treatment is 14 days, so our study requires the intervention group to take medication for at least 7 days. Ginkgolide® was infused intravenously within 24 h after the initiation of alteplase, and the researcher should record the time from thrombolysis to Ginkgolide® use. Patients in hospital which were allocated to the control arm received rt-PA and 250 ml normal saline combined standard-of-care therapy following current clinical guidelines. Patients were followed at 7 and 90 days.

Ginkgolide® was obtained from Chengdu Baiyu Pharmaceutical Company Limited (each 2 ml per vial, containing terpene lactone 10 mg, batch: No.13110002). Intravenous rt-PA treatment was initiated at a standard dose and regimen (0.9 mg/kg, initial bolus of 10% of the final dose and the remaining dose as an intravenous infusion lasting 60 min) following current clinical guidelines. When a patient met all the inclusion/exclusion criteria and signed the informed consent, Ginkgolide® infusion was initiated within 24 h after starting the infusion of rt-PA treatment.

### Outcome Measures

The primary outcome was the proportion of patients with modified Rankin scale (mRS) ≤2 at 90 days. A structured modified Rankin Score at 90 days of AIS patients was followed up with telephone questionnaires by external clinical evaluators who were blinded to the patients’ clinical data. The telephone questionnaire had been validated and was used in previous trial (Collaboration, 2019). The process of telephone assessment was recorded and could be reviewed at any time. Secondary outcomes included National Institute of Health Stroke Scale (NIHSS) scores at 24 h; early neurological improvement (ENI), which was defined as (baseline NIHSS—NIHSS at 7 days)/baseline NIHSS*100%≥18% at 7 days. NIHSS scores were performed by staffs who were not aware of treatment allocation. The mRS scale at 90 days was also followed up with telephone questionnaires by external clinical evaluators who were blinded to the patients’ clinical data. The safety outcomes included any intracranial hemorrhage transformation and symptomatic intracranial hemorrhage on the 7-day follow-up. CT scan was performed within 24 h and on 7 ± 1th day after thrombolysis for assessment of hemorrhage, and additional images might be performed in the case of clinical worsening or at the discretion of the treating physicians. Hemorrhagic transformation was classified into hemorrhagic infarction (HI) and parenchymal hemorrhage (PH). An intracerebral hemorrhage was defined as symptomatic intracranial hemorrhage (sICH) if the patient had clinical deterioration causing an increase of NIHSS ≥4 points and if the hemorrhage was likely to be the cause of the clinical deterioration (Hacke et al., 1998).

### Sample Size

A pre-randomization survey at participating clusters was conducted. According to previous study, the neurological prognosis was increased 21.1% after the treatment of Ginkgolide. Therefore, a total of 894 patients at 14 hospitals (considering a median of 80 AIS patients treated with rt-PA per hospital) would be required to detect a 21% improvement in AIS patients treated with Ginkgolide combined rt-PA (Yuan and Guo, 2017), with 90% power, 5% significance level, and an intra-cluster correlation coefficient (ICC) of 0.05. Taking into account an estimated 20% rate of non-assessable patients, each arm was required to enroll 560 patients. The sample size calculation formula is as follows (Nijders and Bosker, 1999):

$$N={\left(Z_{1-\alpha /2}+Z_{1-\beta }\right)}^{2}*\left[p1\left(1-p1\right)+p0\left(1-p0\right)\right]\;\;\left[1+\left(nj-1\right)\rho I\right]/{\left(p1-p0\right)}^{2}$$

Z 1-α/2 and Z1-β: Z statistic of type I errors and type II errors; p1: Hypothetical rate of primary outcome in the intervention group; p0: Hypothetical rate of primary outcome in the control group; nj: the median number of AIS patients treated with rt-PA per hospital; ρI: intra-cluster correlation coefficient.

### Statistical Analysis

We retrieved demographic and clinical data, the vascular risk factors, time interval from stroke onset to reperfusion therapy (ONT), baseline NIHSS, 24-hour NIHSS and 7-day NIHSS. Clinical outcome at 90 days was assessed with mRS score and dichotomized into good outcome (0–2) and poor outcome (3–6).

Full analysis set analyses were conducted. Per-protocol set (PPS) analyses were conducted: for these analyses we only included completers who received therapy at least 7 days and didn’t experience PH transformation within 24 h after thrombolysis. Our data analyses focused on the predefined primary and secondary outcomes of the trial in our pre-registration (NCT03772847). Fisher’s exact test was used to compare the dichotomous variables between groups, while independent samples two-tailed *t*-test or Mann-Whitney U test was used for the continuous variables, depending on the normality of the distribution. Intra-cluster correlation coefficients (ICC) were calculated using the correlation-based estimation methods for categorical outcomes. To statistically analyze whether there were differences in primary/secondary outcome and safety outcomes between two groups, binary logistic regression analysis was conducted.

Since several baseline factors showed significant differences, we further created a cohort at a 1:1 ratio using propensity score-matching techniques. The use of propensity score analyses balanced the distribution of covariates between treatment and control groups and therefore minimized the influence of potential bias. The resulting propensity score for the treatment of Ginkgolide® included the following 6 variables: age, sex, baseline NIHSS, history of smoking, hypertension, atrial fibrillation. An additional conditional logistic regression was done for primary outcome and safety outcomes by adjusting baseline NIHSS. Odds ratios (OR), 95% confidence intervals (CI), and *p* values were calculated. All tests were two-sided, and statistical significance was set at a probability value of <0.05. All statistical analyses were performed with SPSS 20.0, SAS 9.4 and R 4.0.1 package.

## Results

### Hospital and Patients’ Characteristics

**FIGURE 1:**
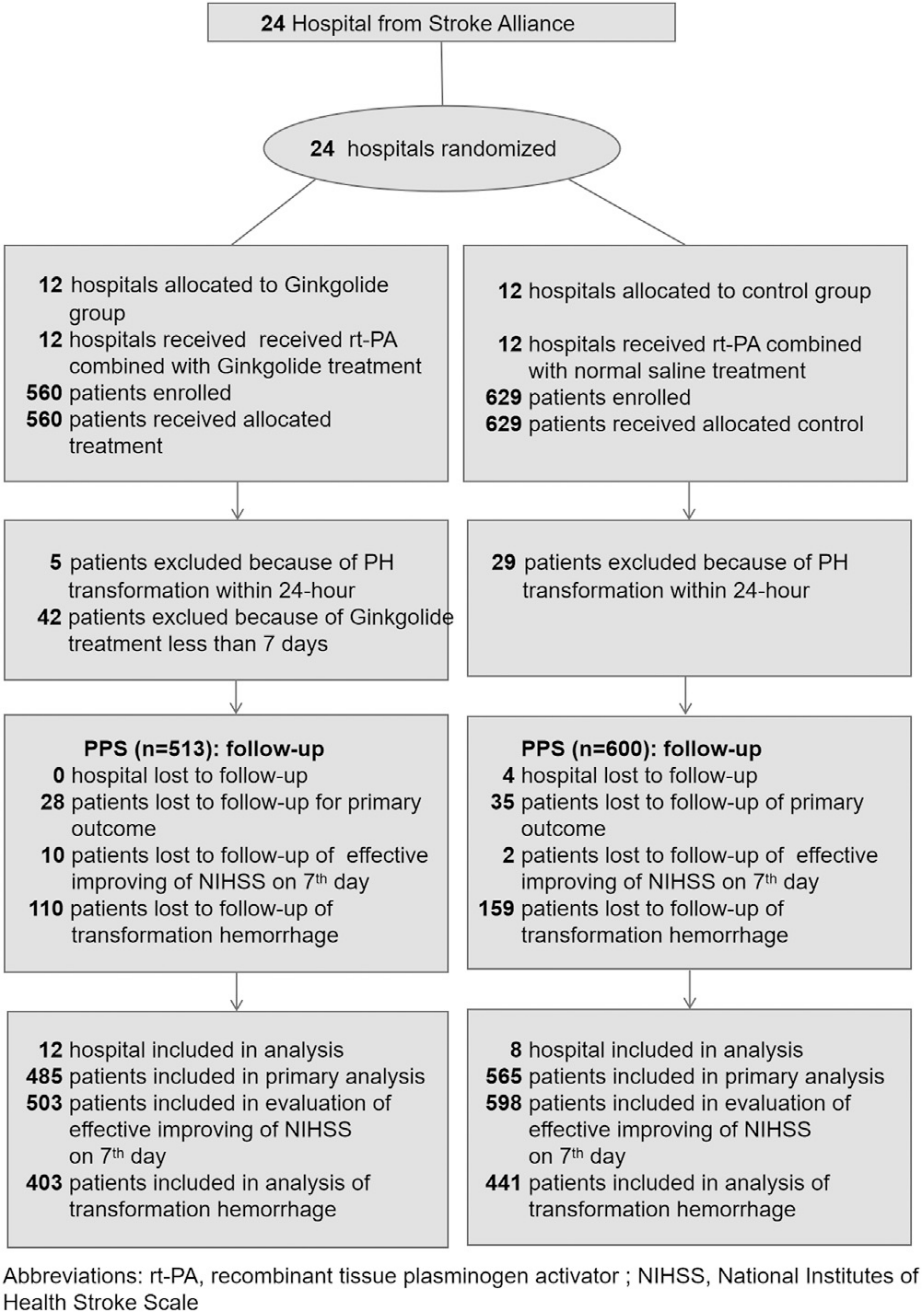
Flow of hospitals and patients through the study.

Twenty-four hospitals were involved in the trial, but 4 hospitals in control group withdrew at the beginning of the study period. 1189 patients fulfilled the inclusion/exclusion criterion. We excluded 29 patients in the control group and 5 patients in the intervention group because of PH transformation within 24-hour after IV alteplase. We excluded 42 patients in the intervention group because they did not receive treatment for at least 7 days. Finally, 1113 patients were enrolled in the PPS analysis. Of these, 513 were in the intervention group and 600 were in the control group. Study design and timeframe including number of enrolled cases are provided in Figure 1.

The median age was 69 years (mean 69 ± 12 years, range 60–78 years), 452 (40.6%) patients were women. The median baseline NIHSS score was 5 (IQR 3–10). The median onset to IVT was 153 min (IQR 108–203 min). The median time from thrombolysis to Ginkgolide use was 115 min (IQR 15–961 min). A total of 758/1050 (72.2%) patients experienced good outcome. Good outcome was achieved in 78.6% patients in the Ginkgolide group and 66.7% in the control group. Follow-up scans after treatment revealed hemorrhage transformation in 66/844 (7.8%) patients, and sICH was observed in 12/844 (1.4%) patients. Baseline characteristics are shown in Table 1. Results of full analysis set analysis are shown in Supplementary Tables S1, S2.

**TABLE 1:** Univariate comparison of characteristics stratified by intervention in unmatched and propensity-matched patients.

	Unmatched		*p* Value	Propensity-matched^a^		*p* Value
	Ginkgolide *n* = 513	Control *n* = 600		Ginkgolide *n* = 404	Control *n* = 404	
Age (year)	67 ± 12	69 ± 13	0.074	68 ± 12	68 ± 12	0.528
Female, n (%)	208 (40.5%)	244 (40.7%)	0.967	252 (62.4%)	240 (59.4%)	0.428
Smoking, n (%)	191 (37.2%)	182 (30.3%)	0.015	153 (37.9%)	143 (35.4%)	0.511
Hypertension, n (%)	351 (68.4%)	365 (60.8%)	0.008	264 (25.3%)	262 (64.9%)	0.941
Diabetes mellitus, n (%)	88 (17.1%)	86 (14.3%)	0.196	65 (16.1%)	65 (16.1%)	1.000
Atrial fibrillation, n (%)	83 (16.2%)	126 (21.0%)	0.040	66 (16.3%)	67 (16.6%)	1.000
Baseline NIHSS	5 (2–9)	5 (3–12)	<0.001	5 (3–9)	4 (2–9)	0.292
24-hour NIHSS	3 (1–6)	4 (1–9)	<0.001	2 (1–5)	3 (1–6)	0.485
Onset-to-needle time (min)	150 (100–205)	156 (112–203)	0.121	148 (98–203)	158 (115–204)	0.280

a This cohot was created at a 1:1 ratio using propensity score-matching techniques for primary outcome of “good outcome at 90 days”.

### Unmatched Analysis

**FIGURE 2:**
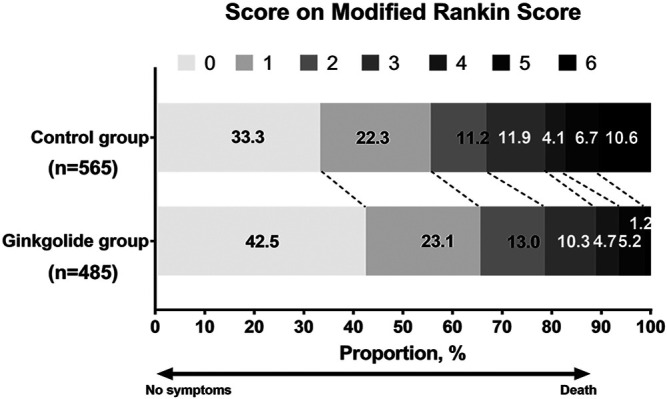
Distribution of Modified Rankin Scores at 90 days Among Eligible Patients with Ginkgolide intervention vs. Control Group.

As Table 1 shows, patients in the Ginkgolide® group had higher rates of smoking (37.2 vs. 30.3%, *p* = 0.015) and hypertension (68.4 vs. 60.8%, *p* < 0.01) than patients in the control group. However, the intervention group had lower baseline NIHSS (5 (2–9) vs. 5 (3–12), *p* < 0.001) and lower rates of atrial fibrillation (16.2 vs. 21.0%, *p* = 0.04). There were no significant differences in other variables. Patients in the Ginkgolide® group were more likely to have good outcomes (78.6 vs. 66.7%, *p* < 0.01) and lower rate of sICH (0 vs. 2.72%, *p* < 0.01), compared with patients in the control group. Figure 2 shows the distribution of mRS values at 90 days. Patients in the Ginkgolide® group were more likely to have early neurological improvement (intervention vs control: 74.0 vs 67.7%, *p* = 0.02). As Table 2 shows, binary logistic regression analysis revealed that the usage of Ginkgolide® was independently associated with good outcome (OR 1.498; 95% CI 1.006–2.029, *p* = 0.009) and early neurological improvement (OR 1.395; 95% CI 1.068–1.814, *p* = 0.014). The usage of Ginkgolide® was also not associated with hemorrhage transformation (OR 0.708; 95% CI 0.412–1.218, *p* = 0.212).

**TABLE 2:** Neurological Outcome and Complication Among Ginkgolide intervention vs. Control Group after binary logistic regression.

Variables	ICC	Ginkgolide group, no. of events/Total patients (%)	Control group, no. of events/Total patients (%)	Odds ratio (95% CI)^a^	*p* Value
Primary outcome					
Good outcome at 90 days, No. (%)	0.033	381/485 (78.6)	377/565 (66.7)	1.498 (1.106–2.029)	0.009
Secondary outcome					
Early neurological improvement, No. (%)	0.002	372/503 (74.0%)	405/598 (67.7%)	1.392 (1.068–1.814)	0.014
Safety outcome					
sICH, No. (%)	0.031	0/403 (0%)	12/441 (2.72%)	-	-
Hemorrhage transformation, No. (%)	0.041	24/403 (6.0%)	42/441 (9.5%)	0.708 (0.412–1.218)	0.212

a Adjusted for age, hypertension, atrial fibrillation, smoking and baseline NIHSS.

### Propensity-Matched Analysis

Propensity score analysis, balanced for age, gender, baseline NIHSS, history of smoking, hypertension, diabetes mellitus and atrial fibrillation, identified 404 matched patient pairs for outcome of “good outcome at 90 days.” As Table 1 shows, all baseline variables were comparable between two groups. Considering the powerful effect of baseline NIHSS on primary outcome, conditional logistic regression was done for outcomes by adjusting baseline NIHSS. Intervention with Ginkgolide® significantly associated with 90-day good outcome (OR 1.513; 95% CI 1.073–2.132, *p* = 0.018) and early neurological improvement (OR 1.574; 95% CI 1.164–2.128, *p* = 0.003) (Table 3). No significant difference in safety outcome of hemorrhage transformation was seen between the 2 matched cohorts (Table 3).

**TABLE 3:** Neurological Outcome and Complication Among matched cohorts between two groups after conditional logistic regression.

Variables^a^	Ginkgolide group, no. of events/Total patients (%)	Control group, no. of events/Total patients (%)	Odds ratio (95% CI)^b^	*p* Value
Primary outcome				
Good outcome at 90 days, No. (%)	311/404 (77.0)	285/404 (70.5)	1.513 (1.073,2.132)	0.018
Secondary outcome				
Early neurological improvement, No. (%) (%)	321/423 (75.9)	282/423 (66.7)	1.574 (1.164,2.128)	0.003
Safety outcome				
sICH, No. (%)	0/318 (0)	5/318 (1.6)	—	—
Hemorrhage transformation, No.(%)	18/318 (5.7)	20/318 (6.3)	0.885 (0.450,1.741)	0.724

a We created different cohorts according to the specific outcome at a 1:1 ratio using propensity score-matching techniques.

b Adjusted for baseline NIHSS.

## Discussion

This cluster-randomized trial showed that Ginkgolide® was effective in improving neurological deficit after rtPA therapy in AIS patients. Additionally, the safety data analysis demonstrated that Ginkgolide® did not increase the incidence of hemorrhage transformation events.

Although mechanical thrombectomy has recently been established as the standard of care for selected patients with large vessel occlusions, less than 10% of all stroke patients are currently eligible for mechanical thrombectomy based on current guidelines. In this regard, intravenous thrombolysis with alteplase remains a viable treatment for the majority of AIS patients in many centers. A number of experimental studies showed the neuroprotective efficacy of Ginkgolide, which can inhibit the platelet aggregation and increase vascular recanalization in AIS patients (Feng et al., 2019; Dong et al., 2021). Ginkgolide played a role as an inhabitation of PAF receptor, which was induced by ischemic stroke (Joseph et al., 1989). The reduction in PAF and its pathway were reportedly helpful to reduce the volume of infarction in acute phase (Oberpichler et al., 1990). A recently clinical trial also confirmed that Ginkgolide helped in decreasing accumulation of PAF after ischemic stroke, which might be one of the mechanisms in reducing stroke recurrence (Dong et al., 2021). Both Ginkgo biloba extract and its constituent ginkgolide were proved effectively attenuating the rtPA-induced disturbances in neurotransmitter, amino acid, energy, lipid, and nucleotide metabolisms (Pietri et al., 1997; Huang et al., 2012; Li et al., 2013; Chen et al., 2018; Feng et al., 2019; Liu et al., 2019). Chen et al. showed that rtPA upregulated the production of glutamic acid, aspartic acid, N-acetyl-l-aspartic acid, and glutamine. Although both diterpene ginkgolide and ginkgo biloba extract ameliorated the upregulation of aspartic acid and glutamine, diterpene ginkgolide also ameliorated the upregulation of glutamic acid and N-acetyl-l-aspartic acid (Chen et al., 2018). Thus, diterpene ginkgolide may exert its neuroprotective effects by reducing the excess production of glutamate and aspartate excitotoxicity, while ginkgo biloba extract may partially ameliorate the excitotoxicity induced by rtPA. But so far, there have been few clinical trials of Ginkgolide. GIANT attained more encouraging results than previous trials because only patients that received thrombolytic therapy were allowed in this study. To the best of our knowledge, this was the largest study to address the management of Ginkgolide® within the first 24 h after IVT.

In our study, 66.7% of rt-PA-treated patients in the control group had good outcome after 3 months, which was in accordance to results in the Thrombolysis Implementation and Monitor of Acute Ischemic Stroke in China (Zhou et al., 2020). In our study, the degree of improvement of the neurological impairment was more pronounced in the Ginkgolide® combined with IVT, compared with the control group, indicating that early use of Ginkgolide® within 24 h after IVT might improve the neurological function of AIS patients. This result was consistent with those of previous studies that showed ginkgolide B and bilobalide might provide neuroprotective effects against rt-PA-induced toxicity.

The antioxidant effects of Ginkgolide® could have contributed to the clinical benefits reported in the trial. Researchers found ginkgolide B reduced reactive oxygen species and restored cerebral blood flow in hyperglycemic rats (Huang et al., 2012). Of note, ginkgolide B treatment could significantly increase the expressions of anti-oxidative stress-related proteins, such as Nrf2. Ginkgolide B was also believed to interfere with the production of free radicals after ischemia (Pietri et al., 1997). These characteristics may support the Ginkgolide® as an antioxidant in AIS patients within the first 24 h after IVT. Furthermore, ginkgolide B could also protect brain from endoplasmic reticulum (ER) stress, which was also an essential signaling event in the progression of brain ischemic/reperfusion injury. In a cell model, preincubation with ginkgolide B could attenuate bupivacaine-induced ER stress and cell apoptosis (Li et al., 2013). Another important component of Ginkgolide®, bilobalide, can also reduce ER stress by increasing the expression of catalase and glutathione (Lu et al., 2016). Hence, the early use of Ginkgolide® after IVT may prevent further oxidative stress injury and ER stress and thereby improve the functional outcome.

The N-methyl-D-aspartate receptor also played a pivotal role in the process of glutamate-induced excitotoxicity in stroke (Wu and Tymianski, 2018). Intravenous rt-PA can potentiate excitotoxic lesions and lead to neuronal death induced by NMDA (Nicole et al., 2001). Administration of Ginkgo biloba extract effectively inhibited NMDA-receptor and ameliorated metabolic disturbances induced by rt-PA (Chen et al., 2018). Moreover, bilobalide enhanced cell viabilities, inhibited apoptosis, and attenuated mitochondrial membrane potential depolarization (Shi et al., 2010; Shi et al., 2011). By triggering various pathways, Ginkgolide® seemed to interrupt the development of pathological processes that lead to ischemic/reperfusion injury after rtPA therapy.

During recent years, several cases of hemorrhage including subdural hematoma (Rowin and Lewis, 1996), subarachnoid hemorrhage (Vale, 1998), intracerebral hemorrhage (Matthews, 1998), have been reported to occur in coincidence with the use of Ginkgo products and those observations have generally been explained by the platelet-activating factor (PAF)-antagonistic action of ginkgolides. However, in this study, ultra-early administration of Ginkgolide® after IVT did not involve a higher risk of hemorrhage transformation or sICH. Indeed, results from different studies consistently indicated that Ginkgo does not significantly affect hemostasis nor the safety of co-administered aspirin, warfarin and other antiplatelet drugs (Bone, 2008). E Koch confirmed that induction of aggregation of human platelets by PAF requires higher concentration, which were generally more than 100 times higher as the peak plasma values measured after oral intake ginkgo biloba extract at recommended doses (Koch, 2005). Therefore, the likelihood of hemorrhage transformation due to PAF is very low, and those case reports might be coincidences. *In vitro* multicellular network model, pretreatment with Ginkgo biloba extract or ginkgolide B enhanced the *trans*-endothelial electrical resistance of capillary endothelial monolayers, reduced the endothelial permeability coefficients for sodium fluorescein, and increased the expression levels of tight junction proteins, namely, ZO-1 and occludin, in endothelial cells (Yang et al., 2017). Results demonstrated the preventive effects of Ginkgo biloba extract on neuronal cell death and enhancement of the function of brain capillary endothelial monolayers after oxygen–glucose deprivation/reoxygenation injury *in vitro*. Ginkgolides® are mainly composed of ginkgo diterpene lactones (ginkgolide A, B, and C) and bilobalide. Ginkgo diterpene lactone mainly plays a role in inhibiting platelet aggregation caused by PAF and inhibiting the production of inflammatory molecules during ischemia-reperfusion, while bilobalide mainly functions to maintain the integrity of vascular endothelial cells and promote vascular endothelial proliferation. Our result also showed the use of Ginkgolide® was not associated with hemorrhage transformation after adjusting baseline NIHSS, indicating that ginkgolides could not increase hemorrhage transformation in AIS patients receiving IV rt-PA, indicating that it is safe and effective to use Ginkgolides® within 24 h after intravenous thrombolysis. It may be due to the anti-platelet aggregation and anti-inflammation of ginkgolides, while bilobalide protects blood brain barrier permeability, which may further diminish the risk of hemorrhage transformation.

Limitations include biased baseline characters such as baseline NIHSS, hypertension and atrial fibrillation, although after adjusting for baseline NIHSS and these comorbidities, intervention with Ginkgolide® was still significantly associated with 90-day mRS. Secondly, the underlying mechanism of Ginkgolide improving neurological deficits was not revealed in our study, which need further imaging or lab markers. Thirdly, we analyzed patients mostly in the Yangtze River Delta, which may introduce geographic bias.

## Conclusion

In summary, the present study suggests that Ginkgolide® use in patients within the first 24 h after IVT was safe and could have a favorable impact on functional outcome. Ginkgolide® therapy might be the treatment of choice for patients at the first 24 h after IVT. Confirmation of these findings in a larger randomized trial is needed.

## GIANT Investigators

Gu Qun, Department of Neurology, The First People's Hospital of Huzhou, Huzhou, China; Wang Yaxian, Department of Neurology, Huzhou Central Hospital, Huzhou, China; Chen Chaochan, Department of Neurology, The First People's Hospital of Yonkang, Jinhua, China; Sui Yi, Department of Neurology, The First People's Hospital of Shenyang, Shenyang, China; Lan Likang, Department of Neurology, Lishui City People's Hospital, Lishui, China; Zhong Jianbin, Department of Neurology, Guangzhou Zengcheng District People’s Hospital, Guangzhou, China; Xu Dongjuan, Department of Neurology, Donyang People's Hospital, Jinhua, China; Hu Haifang, Department of Neurology, The First People's Hospital of Hangzhou Xiaoshan District, Hangzhou, China; Huang Huadong, Department of Neurology, Sahzu.Changxing Campus, Huzhou, China; Cai Xueli, Department of Neurology, Lishui Central Hospital, Lishui, China; Hou Shuangxing, Department of Neurology, ShangHai Pudong Hospital, Shanghai, China; Zhang Ningyuan, Department of Neurology, The First People's Hospital of Tongxiang, Jiaxin, China; Bi Yong, Department of Neurology, ShangHai Fourth People's Hospital, Shanghai, China; Zhang Dechou, Department of Neurology, Affiliated TCM hospital of Southwest Medical University, Sichuan, China; Zhong Lianjiang, Department of Neurology, The Second People's Hospital of Tongxiang, Jiaxin, China;

## Data Availability

The raw data supporting the conclusion of this article will be made available by the authors, without undue reservation.

## Abbreviations

AIS, acute ischemic stroke; BBB, blood brain barrier permeability; CI, confidence intervals; DNA, deoxyribonucleic acid; ENI, early neurological improvement; ER, endoplasmic reticulum; HI, hemorrhagic infarction; ICC, intra-cluster correlation coefficient; IVT, intravenous thrombolysis; IQR, interquartile range; MCAO, middle cerebral artery occlusion; mRS, modified Rankin scale; NIHSS, National Institute of Health Stroke Scale; ONT, onset to reperfusion therapy; OR, odds ratios; PH, parenchymal hemorrhage; PPS, per-protocol set; PAF, platelet-activating factor; rt-PA, recombinant tissue plasminogen activator; SOD, superoxide dismutase; sICH, symptomatic intracranial hemorrhage.

## References

[B1] Bone K. M. (2008). Potential Interaction of Ginkgo Biloba Leaf With Antiplatelet or Anticoagulant Drugs: what Is the Evidence? Mol. Nutr. Food Res. 52, 764–771. 10.1002/mnfr.200700098 18214851

[B2] Chen Z. Bai S. Hu Q. Shen P. Wang T. Liang Z. (2018). Ginkgo Biloba Extract and its Diterpene Ginkgolide Constituents Ameliorate the Metabolic Disturbances Caused by Recombinant Tissue Plasminogen Activator in Rat Prefrontal Cortex. Neuropsychiatr. Dis. Treat. 14, 1755–1772. 10.2147/NDT.S167448 30013348 PMC6037272

[B3] Collaboration F. T. (2019). Effects of Fluoxetine on Functional Outcomes After Acute Stroke (Focus): a Pragmatic, Double-Blind, Randomised, Controlled Trial. Lancet. 393, 265–274. 10.1016/S0140-6736(18)32823-X 30528472 PMC6336936

[B4] Dong Y. Zhang J. Wang Y. Zhao L. Li R. Wei C. GISAA committee (2021). Effect of Ginkgolide in Ischemic Stroke Patients With Large Artery Atherosclerosis: Results From a Randomized Trial. CNS Neurosci. Ther. 00, 1–9. 10.1111/cns.13742 PMC8611772 34676982

[B5] Fang W. Sha L. Kodithuwakku N. D. Wei J. Zhang R. Han D. (2015). Attenuated Blood-Brain Barrier Dysfunction by XQ-1H Following Ischemic Stroke in Hyperlipidemic Rats. Mol. Neurobiol. 52, 162–175. 10.1007/s12035-014-8851-1 25128027

[B6] Feng Z. Sun Q. Chen W. Bai Y. Hu D. Xie X. (2019). The Neuroprotective Mechanisms of Ginkgolides and Bilobalide in Cerebral Ischemic Injury: a Literature Review. Mol. Med. 25 (1), 57. 10.1186/s10020-019-0125-y 31864312 PMC6925848

[B7] Hacke W. Kaste M. Fieschi C. von Kummer R. Davalos A. Meier D. (1998). Randomised Double-Blind Placebo-Controlled Trial of Thrombolytic Therapy With Intravenous Alteplase in Acute Ischaemic Stroke (ECASS II). Second European-Australasian Acute Stroke Study Investigators. Lancet. 352 (9136), 1245–1251. 10.1016/s0140-6736(98)08020-9 9788453

[B8] Huang M. Qian Y. Guan T. Huang L. Tang X. Li Y. (2012). Different Neuroprotective Responses of Ginkgolide B and Bilobalide, the Two Ginkgo Components, in Ischemic Rats With Hyperglycemia. Eur. J. Pharmacol. 677, 71–76. 10.1016/j.ejphar.2011.12.011 22197649

[B9] Hussein H. M. Saleem M. A. Qureshi A. I. (2018). Rates and Predictors of Futile Recanalization in Patients Undergoing Endovascular Treatment in a Multicenter Clinical Trial. Neuroradiology. 60 (5), 557–563. 10.1007/s00234-018-2016-2 29574600

[B10] Jiang M. Li J. Peng Q. Liu Y. Liu W. Luo C. (2014). Neuroprotective Effects of Bilobalide on Cerebral Ischemia and Reperfusion Injury Are Associated With Inhibition of Pro-inflammatory Mediator Production and Down-Regulation of JNK1/2 and P38 MAPK Activation. J. Neuroinflammation. 11, 167. 10.1186/s12974-014-0167-6 25256700 PMC4189683

[B11] Joseph R. Welch K. M. D'Andrea G. (1989). Effect of Therapy on Platelet Activating Factor-Induced Aggregation in Acute Stroke. Stroke. 20 (5), 609–611. 10.1161/01.str.20.5.609 2718200

[B12] Knecht T. Borlongan C. Dela Peña I. (2018). Combination Therapy for Ischemic Stroke: Novel Approaches to Lengthen Therapeutic Window of Tissue Plasminogen Activator. Brain Circ. 4 (3), 99–108. 10.4103/bc.bc_21_18 30450415 PMC6187940

[B13] Koch E. (2005). Inhibition of Platelet Activating Factor (PAF)-Induced Aggregation of Human Thrombocytes by Ginkgolides: Considerations on Possible Bleeding Complications After Oral Intake of Ginkgo Biloba Extracts. Phytomedicine. 12 (1-2), 10–16. 10.1016/j.phymed.2004.02.002 15693702

[B14] Li L. Zhang Q. G. Lai L. Y. Wen X. J. Zheng T. Cheung C. W. (2013). Neuroprotective Effect of Ginkgolide B on Bupivacaine-Induced Apoptosis in SH-Sy5y Cells. Oxid Med. Cell Longev. 2013, 159864. 10.1155/2013/159864 24228138 PMC3818975

[B15] Li P. Stetler R. A. Leak R. K. Shi Y. Li Y. Yu W. (2018). Oxidative Stress and DNA Damage After Cerebral Ischemia: Potential Therapeutic Targets to Repair the Genome and Improve Stroke Recovery. Neuropharmacology. 134, 208–217. 10.1016/j.neuropharm.2017.11.011 29128308 PMC5940593

[B16] Liu Q. Jin Z. Xu Z. Yang H. Li L. Li G. (2019). Antioxidant Effects of Ginkgolides and Bilobalide Against Cerebral Ischemia Injury by Activating the Akt/Nrf2 Pathway *In Vitro* and *In Vivo* . Cell Stress Chaperones. 24 (2), 441–452. 10.1007/s12192-019-00977-1 30815818 PMC6439064

[B17] Lu L. Wang S. Fu L. Liu D. Zhu Y. Xu A. (2016). Bilobalide Protection of Normal Human Melanocytes from Hydrogen Peroxide-Induced Oxidative Damage via Promotion of Antioxidase Expression and Inhibition of Endoplasmic Reticulum Stress. Clin. Exp. Dermatol. 41, 64–73. 10.1111/ced.12664 26178968

[B18] Matthews M. K. JR. (1998). Association of Ginkgo Biloba With Intracerebral Hemorrhage. Neurology. 50, 1933–1934. 10.1212/wnl.50.6.1933 9633781

[B19] Nicole O. Docagne F. Ali C. Margaill I. Carmeliet P. MacKenzie E. T. (2001). The Proteolytic Activity of Tissue-Plasminogen Activator Enhances NMDA Receptor-Mediated Signaling. Nat. Med. 7 (1), 59–64. 10.1038/83358 11135617

[B20] Nijders S. T. A. B. Bosker R. J. (1999). Multilevel Analysis. 1st ed. New Delhi: SAGE.

[B21] Oberpichler H. Sauer D. Rossberg C. Mennel H. D. Krieglstein J. (1990). PAF Antagonist Ginkgolide B Reduces Postischemic Neuronal Damage in Rat Brain Hippocampus. J. Cereb. Blood Flow Metab. 10 (1), 133–135. 10.1038/jcbfm.1990.17 2298830

[B22] Pietri S. Maurelli E. Drieu K. Culcasi M. (1997). Cardioprotective and Anti-Oxidant Effects of the Terpenoid Constituents of Ginkgo Biloba Extract (EGb 761). J. Mol. Cell Cardiol. 29 (2), 733–742. 10.1006/jmcc.1996.0316 9140830

[B23] Rowin J. Lewis S. L. (1996). Spontaneous Bilateral Subdural Hematomas Associated With Chronic Ginkgo Biloba Ingestion. Neurology. 46 (6), 1775–1776. 10.1212/wnl.46.6.1775 8649594

[B24] Shi C. Wu F. Yew D. T. Xu J. Zhu Y. (2010). Bilobalide Prevents Apoptosis through Activation of the PI3K/Akt Pathway in SH-Sy5y Cells. Apoptosis. 15, 715–727. 10.1007/s10495-010-0492-x 20333467

[B25] Shi C. Zou J. Li G. Ge Z. Yao Z. Xu J. (2011). Bilobalide Protects Mitochondrial Function in Ovariectomized Rats by Up-Regulation of mRNA and Protein Expression of Cytochrome C Oxidase Subunit I. J. Mol. Neurosci. 45, 69–75. 10.1007/s12031-010-9388-z 20490713

[B26] Takagi T. Kitashoji A. Iwawaki T. Tsuruma K. Shimazawa M. Yoshimura S. (2014). Temporal Activation of Nrf2 in the Penumbra and Nrf2 Activator-Mediated Neuroprotection in Ischemia-Reperfusion Injury. Free Radic. Biol. Med. 72, 124–133. 10.1016/j.freeradbiomed.2014.04.009 24746614

[B27] Vale S. (1998). Subarachnoid Haemorrhage Associated With Ginkgo Biloba. Lancet. 352, 36. 10.1016/S0140-6736(05)79516-7 9800751

[B28] Wei J. Fang W. Sha L. Han D. Zhang R. Hao X. (2013). XQ-1H Suppresses Neutrophils Infiltration and Oxidative Stress Induced by Cerebral Ischemia Injury Both *In Vivo* and *In Vitro* . Neurochem. Res. 38, 2542–2549. 10.1007/s11064-013-1176-z 24122081

[B29] William J. Rabinstein Alejandro A. Ackerson T. Adeoye Opeolu M. Bambakidis Nicholas C. Becker K. (2019). Guidelines for the Early Management of Patients With Acute Ischemic Stroke: 2019 Update to the 2018 Guidelines for the Early Management of Acute Ischemic Stroke: A Guideline for Healthcare Professionals From the American Heart Association/American Stroke Association. Stroke. 50 (12), e344–e418. 10.1016/j.phymed.2004.02.002 31662037

[B30] Wu Q. J. Tymianski M. (2018). Targeting NMDA Receptors in Stroke: New Hope in Neuroprotection. Mol. Brain. 11, 15. 10.1186/s13041-018-0357-8 29534733 PMC5851248

[B31] Yang X. Zheng T. Hong H. Cai N. Zhou X. Sun C. (2017). Neuroprotective Effects of Ginkgo Biloba Extract and Ginkgolide B Against Oxygen-Glucose Deprivation/Reoxygenation and Glucose Injury in a New *In Vitro* Multicellular Network Model. Front. Med. 12, 307–318. 10.1007/s11684-017-0547-2 29058254

[B32] Yuan L. F. Guo H. L. (2017). Effect and Mechanism of Ginkgolide Injection Combined with Edaravone on Acute Cerebral Infarction. Chin. J. Prim. Med. Pharm. 24 (18), 2820–2823.

[B33] Yumiko N. Toru Y. Qian L. Kota S. Yasuyuki O. Ryuta M. (2017). Time-dependent Change of *In Vivo* Optical Imaging of Oxidative Stress in a Mouse Stroke Model. J. Neurosci. Res. 95, 2030–2039. 28276088 10.1002/jnr.24047

[B34] Zhao W. Wu C. Dornbos D. 3rd Li S. Song H. Wang Y. (2020). Multiphase Adjuvant Neuroprotection: A Novel Paradigm for Improving Acute Ischemic Stroke Outcomes. Brain Circ. 6 (1), 11–18. 10.4103/bc.bc_58_19 32166195 PMC7045534

[B35] Zhou H. Y. Chen W. Q. Pan Y. S. Suo Y. Meng X. Li H. (2020). Effect of Sex Differences on Prognosis of Intravenous Thrombolysis: Data From the Thrombolysis Implementation and Monitor of Acute Ischemic Stroke in China (TIMS-China). Stroke Vasc. Neurol. 6 (1), 1015 svn-2020-000351. 10.1136/svn-2020-000351 PMC8005908 32641445

