# Supplementary material for: Ginkgolide With Intravenous Alteplase Thrombolysis in Acute Ischemic Stroke Improving Neurological Function: A Multicenter, Cluster-Randomized Trial (GIANT)
Source: Front Pharmacol. 2021 Dec 3;12:792136. doi: 10.3389/fphar.2021.792136 (PMC8681856; doi:10.3389/fphar.2021.792136)
Supplement: Supplementary file 1 [file DataSheet1.docx]

**Supplementary Table 1. Univariate comparison of characteristics stratified by intervention in full analysis.**

|  | **Unmatched** | | ***P* value** |
| --- | --- | --- | --- |
|  | **Ginkgolide**  **n=560** | **Control**  **n=629** |  |
| **Age (year)** | 68$\pm$12 | 69$\pm$13 | 0.213 |
| **Female, n (%)** | 228 (40.7%) | 258 (41.0%) | 0.953 |
| **Smoking, n (%)** | 208 (37.1%) | 189 (30.0%) | 0.010 |
| **Hypertension, n (%)** | 384 (68.6%) | 387 (61.5%) | 0.013 |
| **Diabetes mellitus, n (%)** | 96 (17.1%) | 92 (14.6%) | 0.265 |
| **Atrial fibrillation, n (%)** | 101 (18.0%) | 134 (21.3%) | 0.166 |
| **Baseline NIHSS** | 5 (3-10) | 6 (3-12) | 0.001 |
| **24-hour NIHSS** | 3 (1-7) | 4 (1-10) | 0.001 |
| **Onset-to-needle time (min)** | 150 (101-206) | 155 (112.5-202.5) | 0.036 |

**Supplementary Table 2 Neurological Outcome and Complication Among Acute Ischemic Stroke with Reperfusion therapy Receiving Ginkgolide intervention vs Control Group after binary logistic regression in full analysis.**

| **Variables** | **Ginkgolide group,**  **No. of Events/Total**  **Patients (%)** | **Control group,**  **No. of Events/Total**  **Patients (%)** | **Odds Ratio**  **(95% CI) ^a^** | ***P* value** |
| --- | --- | --- | --- | --- |
| **Primary outcome** |  |  |  |  |
| Good outcome at 90 days, No. (%) | 381/532 (71.6) | 379/594 (63.8) | 1.237 (0.931-1.645) | 0.143 |
| **Secondary outcome** |  |  |  |  |
| Early neurological improvement, No. (%) | 385/546 (70.5%) | 413/627 (65.9%) | 1.245 (0.969-1.599) | 0.087 |
| **Safety outcome** |  |  |  |  |
| sICH, No. (%) | 6/429 (1.4%) | 19/461 (4.1%) | 0.344 (0.134,0.883) | 0.026 |
| Hemorrhage transformation, No. (%) | 37/429 (8.6%) | 60/461 (13.0%) | 0.698 (0.443,1.099) | 0.121 |
